# Feeding of a high protein, low carbohydrate diet leads to greater postprandial energy expenditure and fasted n6: n3 fatty acid ratio in lean, adult dogs compared to a moderate protein, moderate carbohydrate diet

**DOI:** 10.1093/tas/txaf018

**Published:** 2025-02-06

**Authors:** Sydney Banton, Júlia G Pezzali, Taylor Richards, Lyn M Hillyer, David W L Ma, Jesús M Pisco, James R Templeman, Anna K Shoveller

**Affiliations:** Department of Animal Biosciences, University of Guelph, Guelph, ON N1G 2W1, Canada; Department of Animal Biosciences, University of Guelph, Guelph, ON N1G 2W1, Canada; Department of Animal Biosciences, University of Guelph, Guelph, ON N1G 2W1, Canada; Department of Human Health & Nutritional Sciences, University of Guelph, Guelph, ON N1G 2W1, Canada; Department of Human Health & Nutritional Sciences, University of Guelph, Guelph, ON N1G 2W1, Canada; Department of Animal Biosciences, University of Guelph, Guelph, ON N1G 2W1, Canada; Department of Animal Biosciences, University of Guelph, Guelph, ON N1G 2W1, Canada; Department of Animal Biosciences, University of Guelph, Guelph, ON N1G 2W1, Canada

**Keywords:** canine, energy expenditure, fatty acids, high protein

## Abstract

High protein, low carbohydrate (HPLC) diets are often sought out by dog owners. They are reported to have beneficial effects on energy expenditure (EE), fat oxidation, and may alter the serum fatty acid profile. However, there is little to no data to support the feeding of HPLC diets to healthy adult dogs. Thus, the objective of the present study was to interrogate the health claims related to the feeding of HPLC diets to healthy adult dogs using a commercially available HPLC diet (48% of metabolizable energy (ME) from protein, 10% of ME from nitrogen-free extract; NFE), a moderate protein, moderate carbohydrate (MPMC) diet (28% of ME from protein, 39% of ME from NFE) formulated with the same ingredients as HPLC, and a commercially available, MPMC, high fiber, “metabolic” (MET) diet (30% of ME from protein, 37% of ME from NFE) as a commercial control. Diets were fed to 9 healthy, large breed dogs for 42 d in a Latin square design. Fasted blood samples were collected on days −2 (baseline), 12, 26 and 40, and indirect calorimetry was performed on 8 dogs on days 20 and 39 to measure respiratory quotient (RQ) and EE. Statistics were performed in SAS Studio (version 9.4). Dogs fed HPLC had a lower RQ at fasted (0.76), 0 to 4 h (0.78) and 5 to 10 h (0.83) post-meal compared to dogs fed MET (0.80, 0.83, 0.90) and MPMC (0.80, 0.84, 0.91; P < 0.05). All dogs had a similar EE at fasted but dogs fed HPLC had a greater postprandial EE at 0 to 4 h (5.36 kcal/kg^0.75^) and 5 to 10 h (5.46 kcal/kg^0.75^) compared to dogs fed MPMC (4.79 and 4.84 kcal/kg^0.75^; P < 0.05). Dogs fed MET (4.98, 4.96 kcal/kg^0.75^) were similar to both (P > 0.05). Alpha-linolenic acid (ALA; 18:3n3), docosapentaenoic acid (DPA; 22:5n3) and total n3 were all greater in dogs fed MET at each week (P < 0.05). In contrast, total n6: n3 and arachidonic acid (ARA; 20:4n6): eicosapentaenoic acid (EPA; 20:5n3) were both greater (P < 0.05) in dogs fed HPLC and MPMC compared to dogs fed MET at each week. This study represents the first to assess EE and serum fatty acids in adult large breed dogs consuming a HPLC diet for 6 wk. Consuming a HPLC diet led to potential beneficial effects of increased EE and fat oxidation after a meal, which has the potential to be useful in managing obesity, a common health concern in dogs.

## Teaser

This study suggests that dogs fed a high protein, low carbohydrate diet have increased energy expenditure and fat oxidation after a meal in comparison to dogs fed a moderate protein, moderate carbohydrate diet. This suggests the potential for high protein, low carbohydrate diets to aid in weight management if the diet were to be appropriately calorie-restricted.

## INTRODUCTION

High protein, low carbohydrate (HPLC) diets have become increasingly popular among dog owners as people continue to want to feed their dogs similarly to how they feed themselves. During a state of low carbohydrate intake, glucose availability is lower and the rate of fatty acid mobilization from adipose tissue is higher to supply energy to peripheral tissues (reviewed in Newman and Verdin, 2017). Beta-oxidation of fatty acids in the mitochondria of hepatocytes results in acetyl CoA as the end product, which is converted to ketone bodies, with β-hydroxybutyrate (BOHB) being the most abundant produced in mammals (reviewed in Newman and Verdin, 2017). Ketone bodies can then be exported out of the liver and used for energy by other tissues. Although often poorly defined, the general consensus of what constitutes a HPLC diet in humans is at least 25% to 30% of energy coming from protein (Westerterp-Plantenga et al., 2012) and ≤30% of energy coming from carbohydrates (Chacón et al., 2024). However, in a recent analysis of dog foods available in the United States, the average protein content was 29% and carbohydrate content was 50% on a dry matter basis (Wall, 2019). Therefore, a high protein dog diet would likely be higher than what is considered high protein for humans. High protein diets have been extensively studied in human nutrition (Johnston et al., 2006; Forsythe et al., 2008; Ebbeling et al., 2012; Martens et al., 2015), but their macronutrient content varies. High protein, low carbohydrate diets maintain total EE and can lead to a greater amount of fat oxidation after weight loss in overweight young people compared to when low fat, high carbohydrate (Ebbeling et al., 2012) or low protein, high carbohydrate diets (Martens et al., 2015) are consumed. Although HPLC diets are widely available to dog owners today (Phillips-Donaldson, 2018), none of these outcomes have been studied in dogs.

Because of the reduced carbohydrate content, HPLC diets can be higher in fat, often referred to as “ketogenic” diets, and can increase high- and low-density lipoprotein cholesterol (HDL and LDL, respectively; Mansoor et al., 2016), the latter of which is known to be associated with cardiovascular disease, especially atherosclerosis, in humans (Vekic et al., 2022). However, dogs have a greater proportion of HDL cholesterol compared to humans (Ramaswamy et al., 1999), and atherosclerosis is rare in dogs and often associated with diabetes and hypothyroidism rather than LDL cholesterol (Hess et al., 2003). Additionally, in contrast to a low carbohydrate diet that increases β-oxidation of fatty acids, a high carbohydrate diet is thought to decrease β-oxidation and increase de novo fatty acid synthesis (Parks, 2002). Thus, another reason why HPLC diets are of interest in human nutrition is their reported ability to favorably alter circulating fatty acids in those with metabolic syndrome. In one study of overweight humans with elevated triglycerides and low HDL cholesterol, those consuming an energy-restricted, very low carbohydrate (12%), high protein (28%), high fat (59%) diet had an elevated serum n6: n3 and a greater arachidonic acid (ARA; 20:4n6): eicosapentaenoic acid (EPA; 20:5n3) compared to those consuming a moderate carbohydrate (56%), moderate protein (20%), low fat (24%) diet for 12 wk (Forsythe et al., 2008). Similarly, this increase in the ARA: EPA has been reported in overweight adults consuming an energy-restricted ketogenic, high fat (60% of energy), high protein (31% of energy), very low carbohydrate (9% of energy) diet compared to a low fat (30% of energy), high protein (28% of energy), low carbohydrate (42% of energy) diet after 6 wk (Johnston et al., 2006). To the author’s knowledge, only one study has assessed the circulating fatty acids in healthy adult dogs fed a HPLC diet and also reported a greater n6: n3 ratio in dogs fed HPLC (53% of ME from protein, 8% of ME from carbohydrate) compared to a high fat (69% of ME), low carbohydrate (5% of ME) and moderate protein, moderate carbohydrate (MPMC) diet (Jackson, 2022). However, the serum fatty acid profiles of healthy humans or dogs consuming low carbohydrate diets is scarce. Given that obesity is a growing concern in the canine population, the consumption of HPLC diets and the reported increase in EE and altered circulating fatty acids may be beneficial for weight maintenance in lean, healthy dogs.

Thus, the objective of the present study was to investigate EE and serum fatty acid and BOHB concentrations in healthy adult dogs fed either a commercially available HPLC diet (48% of metabolizable energy (ME) from protein, 10% of ME from nitrogen-free extract; NFE), a MPMC diet (28% of ME from protein, 39% of ME from NFE) formulated with the same ingredients as HPLC, and a commercially available, high fiber, MPMC, “metabolic” (MET) diet (30% of ME from protein, 37% of ME from NFE) as a commercial control. We hypothesized that dogs consuming HPLC would have greater postprandial EE and respiratory quotient (RQ), a greater serum n6: n3 and ARA: EPA, and greater BOHB concentrations compared to dogs consuming MPMC and MET. We also sought to investigate the overall implications on the health of dogs consuming HPLC for 6 wk via complete blood count and serum biochemistry.

## MATERIALS AND METHODS

### Animals and Housing

All experimental procedures were approved by the University of Guelph’s Animal Care Committee (AUP #4646). Nine adult, male-neutered, healthy mixed-breed hounds [1.8 ± 0.1 yr, 26.1 ± 0.9 kg, 8/9 dogs with a body condition score (BCS) of 4 to 5 and 1 with a BCS of 6 on a 9-point scale (Laflamme, 1997)] were housed at the Central Animal Facility at the University of Guelph. Dogs were housed in kennels (7.4 square meters) either individually with nose-to-nose contact or pair-housed (two pairs). The rooms were maintained at 21 °C with a relative humidity of 50% to 60% and a 12:12 (L:D)h schedule with lights on from 0700 h to 1900 h. Dogs had unlimited access to rubber and nylon toys and water within each kennel. All dogs were walked outdoors for 20 min, 6 d/wk, unless weather was poor, in which case dogs were exercised in an indoor playroom for 20 min.

### Diets and Study Design

The diets and design of this study was the same as Banton et al. (2025). Briefly, a commercial HPLC diet (Ketona Chicken Recipe Dog Food, Ketonatural Pet Foods, Salt Lake City, UT), an experimental MPMC diet formulated with the same ingredients as HPLC, and a commercial extruded MPMC, high fiber, “metabolic” diet (Metabolic Chicken Flavor Dog Food, Hill’s Prescription Diet, Topeka, KS) were used (Table 1). The MET diet is intended for weight loss, if calorie-restricted, but can also be fed to maintenance requirements, and to improve satiety via increased TDF. The TDF was measured via the AFVAN-SLMF-0017 method, which uses three separate analyses for TDF, insoluble fiber and soluble fiber, not by calculating the difference. Therefore, the insoluble fiber can have a >100% recovery and be greater than TDF. The MET diet was used in this study as a positive control. The MPMC diet was formulated to meet similar macronutrient targets as MET, but using the same ingredients as HPLC. A portion of the protein sources (chicken and pea) in the HPLC diet was replaced with brown rice in the MPMC diet (Table 2). Both HPLC and MPMC contained supplemental vitamins, minerals and choline chloride. The MET diet contained supplemental vitamins, minerals, DL-methionine, taurine, L-lysine, choline chloride and L-carnitine. Each dog was fed each diet for 42 d in a complete, randomized, replicated 3 × 3 Latin square design. The dogs were fed a wash-in diet (S6 Nutram Sound Balanced Wellness Adult, Nutram Pet Products, Elmira, ON) for 14 d before and between each of the experimental periods.

**Table 1.** Nutrient analysis and energy content of the high protein, low carbohydrate (HPLC), moderate protein, moderate carbohydrate (MPMC) and metabolic (MET) diet on an as-fed basis

	HPLC	MPMC	MET
**Proximate analysis, %**			
Moisture	7.59	8.12	9.93
Crude protein (CP)	52.1	28.7	27.2
Crude fat	19.0	14.5	12.5
Crude Fiber (CF)	3.28	3.04	10.2
Total dietary fiber (TDF)	7.0	7.9	16.0
Insoluble	10.9	8.4	16.5
Soluble	1.1	0.8	1.7
Calculated nitrogen-free extract (NFE_CF_^1^)	10.7	40.0	34.2
Calculated NFE_TDF_^2^	6.9	35.2	28.4
Ash	7.38	5.63	6.01
**Energy content**			
Calculated metabolizable energy (ME_AAFCO_)^3^, kcal/kg	3811	3637	3210
Crude protein (% of ME)	47.8	27.6	29.7
Crude fat (% of ME)	42.4	33.9	33.1
NFE (% of ME)	9.8	38.5	37.3
Calculated ME_NRC_CF_^4^, kcal/kg	4046	3821	3127
Calculated ME_NRC_TDF_^5^, kcal/kg	4221	3946	3416
Ketogenic ratio^6^	0.96	0.45	0.46
**IDAA, %**			
Arginine	3.25	1.76	1.25
Histidine	1.14	0.57	0.47
Isoleucine	1.91	1.06	0.86
Leucine	3.39	1.79	2.02
Lysine	3.46	1.65	1.40
Methionine	1.09	0.60	0.99
Phenylalanine	1.85	1.00	1.00
Threonine	1.83	0.93	0.81
Tryptophan	0.53	0.28	0.22
Valine	2.06	1.16	1.00
**Fatty acids, g/100 g** ^7^			
Lauric acid (12:0)	0	0	1.58
Myristic acid (14:0)	0.14	0.11	0.87
Pentadecylic acid (15:0)	0.02	0.01	0
Palmitic acid (16:0)	4.54	3.18	2.14
Margaric acid (17:0)	0.03	0.024	0.021
Stearic acid (18:0)	1.29	0.83	0.90
Arachidic acid (20:0)	0.02	0.02	0.02
Total saturated fatty acids	6.04	4.17	5.53
Palmitoleic acid (16:1c9)	1.14	0.77	0.23
Oleic acid (18:1c9)	7.12	5.66	3.35
Gondoic acid (20:1c11)	0.06	0.05	0.05
Total monounsaturated fats	8.32	6.48	3.63
Linoleic acid (18:2n6)	3.67	3.25	2.10
Gamma-linolenic acid (18:3n6)	0.04	0.03	0
Alpha-linolenic acid (18:3n3)	0.42	0.64	0.28
Eicosadienoic acid (20:2n6)	0.04	0.02	0.03
Dihomo-gamma linolenic acid (20:3n6)	0.06	0.03	0.01
Arachidonic acid (20:4n6)	0.22	0.08	0.04
Docosapentaenoic acid (22:5n3)	0.02	0.01	0
Docosahexaenoic acid (22:6n3)	0.01	0	0
Total polyunsaturated fats	4.48	3.70	2.82
Total n-3 fatty acids	0.46	0.29	0.64
Total n-6 fatty acids	4.03	3.41	2.18
n6:n3 ratio	8.76	11.76	3.41
**Other**			
Choline chloride, mg/kg	3020	2570	2380
L-carnitine, mg/kg	27.4	20.6	235

^1^NFE (%) = 100 −  (moisture + crude protein + crude fat + crude fiber + ash).

^2^NFE (%) = 100 − (moisture + crude protein + crude fat + total dietary fiber + ash).

^3^ME_AAFCO_ (kcal/kg) = ((8.5 kcal ME × g crude fat) + (3.5 kcal ME × g crude protein) + (3.5 kcal ME × g nitrogen-free extract) × 10).

^4^ ^,5^ME_NRC_CF, NRC_TDF_ (kcal/kg) = DE  —(1.04 × protein (g))*1000, where DE (kcal/g) = (GE × (ED/100)), where ED (%) = 91.2 –  (1.43 × CF in DM(%)) or ED (%) = 96.6 –  (0.95 × TDF in DM(%))^5^, where GE (kcal/g) = (5.7 × protein (g)) + (9.4 × fat(g)) + (4.1 × (NFE(g) + CF(g))).

^6^Calculated as (0.9 × g of fat) + (0.46 × g of protein) / g of carbohydrate + (0.1 × g of fat) + (0.58 × g of protein)] according to Zilberter & Zilberter (2018).

^7^Fatty acids that were <0.01% in all diets were removed from the table.

**Table 2.** Ingredient inclusion (%) of the high protein, low carbohydrate (HPLC), moderate protein, moderate carbohydrate (MPMC) and ingredient list of the metabolic (MET) diet^1^

Ingredient Name	HPLC	MPMC
Brown rice	-	55.78
Chicken, spray-dried	46.97	26.32
Whole green peas	8.75	-
Pea protein (50% min)	8.75	-
Ground chicken	7.90	-
Oat fiber	7.28	8.47
Chicken meal	6.25	-
Chicken fat	5.05	-
Flax meal	3.30	3.84
Gelatin (porcine)	2.11	2.45
Animal digest, dry	1.00	-
Calcium carbonate	0.75	1
Potassium chloride, 50%	0.45	0.52
Sea salt	0.45	0.52
Choline chloride, 60%	0.19	0.22
Vitamin C, 35%	0.17	0.18
Mineral premix	0.16	0.16
Naturox plus®	0.15	0.18
Citric acid, 99.5%	0.15	0.18
Vitamin premix	0.15	0.14
Albion chelate	0.02	0.02

^1^Whole Grain Wheat, Whole Grain Corn, Chicken Meal, Powdered Cellulose, Soybean Meal, Corn Gluten Meal, Dried Beet Pulp, Dried Tomato Pomace, Hydrolyzed Chicken Flavor, Chicken Fat, Flaxseed, Coconut Oil, Lactic Acid, DL-Methionine, L-Lysine, Carrots, Potassium Chloride, Iodized Salt, Lipoic Acid, vitamins (Vitamin E Supplement, L-Ascorbyl-2-Polyphosphate (source of Vitamin C), Niacin Supplement, Thiamine Mononitrate, Vitamin A Supplement, Calcium Pantothenate, Riboflavin Supplement, Biotin, Vitamin B12 Supplement, Pyridoxine Hydrochloride, Folic Acid, Vitamin D3 Supplement), Choline Chloride, minerals (Manganese Sulfate, Ferrous Sulfate, Zinc Oxide, Copper Sulfate, Calcium Iodate, Sodium Selenite), Taurine, L-Carnitine, Mixed Tocopherols for freshness, Natural Flavors, Beta-Carotene.

Dogs were split into two groups (n = 4 in Group 1 and n = 5 in Group 2) and started each period one day apart from each other in order to accommodate for calorimetry days. Each of the three diets was represented at least once in each group. Dogs were fed to maintain body weight (BW) based on historical feeding records and 2.5% of their calories came from treats used for training (Beef Tendersticks, Crumps’ Naturals, Brampton, ON). Food was mixed with 200 mL of water and offered once daily at 0745 h. These dogs participated in many Indicator Amino Acid Oxidation studies to assess bioavailability. For these diets, they had kibble top-dressed with AA solutions. In order to keep the dogs used to receiving “solutions” with a meal, they were always fed water with their kibble. Dogs were weighed weekly with food intake adjusted as needed in order to maintain BW.

### Blood Collection

Fasted blood samples (3 mL) were collected via cephalic venipuncture on days −2 (baseline), 12, 26 and 40 into a red-top serum tube (Becton Dickinson Canada Inc., Mississauga, ON). Samples were allowed to clot for 30 min and then centrifuged at 2,500 × g for 15 min at 4 °C. Serum was separated and 0.5 mL was sent to the Animal Health Laboratory (AHL) at the University of Guelph for BOHB analysis using the Cobas c501 biochemistry analyzer (Roche Diagnostics International AG, Rotkreuz, Switzerland) following the Randox BHBA kit method (Randox Laboratories Limited, County Antrim, Ireland). This UV method is based on the oxidation of D-3-Hydroxybutyrate to acetoacetate by the enzyme 3-Hydroxybutyrate dehydrogenase. The D-3-Hydroxybutyrate concentration is correlated with the absorbance. The lower limit for detection of D-3-Hydroxybutyrate was 0.100 mmol/L. The remaining serum was frozen at -80 °C for fatty acid analysis.

On days -2 and 40, an additional 2 mL was collected into the red-top serum tube and 0.5 mL into a purple-top EDTA tube (Becton Dickinson Canada Inc.), respectively, for serum biochemistry and HDL and complete blood count (CBC) analysis. These samples were sent to AHL for immediate analysis. Complete blood count was analyzed via an automated hematology system (Advia 2120; Siemens Global, Munich, Germany). The differential was confirmed using a stained blood smear. Serum biochemistry was analyzed via an automated biochemical analyzer (Cobas 6000 c501 Analyzer; Roche Diagnostics). HDL was analyzed via the Cobas HDL-Cholesterol Gen.4 method (Roche Diagnostics).

### Indirect Calorimetry

Indirect calorimetry was performed on days 20 and 39. Only 8 dogs performed indirect calorimetry due to there only being 4 chambers. Dogs were walked to a separate building and placed in calorimetry chambers (76 in × 53 in × 61 in, L × W × H) at ~ 0630 h and remained in the chambers for 12 h. At ~1200 h, calorimetry was paused and dogs were taken outside and given the opportunity to urinate and defecate. They were brought back inside and calorimetry resumed at ~1230 h. Once all dogs were in the chambers in the morning, the system was turned on immediately to begin pulling air. The indirect calorimetry system used was an open circuit, ventilated calorimeter with room air being drawn from each chamber at a rate of ~21 to 35 L/min, depending on the dog to ensure the levels of CO_2_ in each chamber did not go over 10,100 ppm. The exiting chamber air was dried by passing it through cylinders of calcium sulfate anhydrous (Drierite, W.A. Hammond Drierite Company LTD, Xenia, OH) and magnesium perchlorate (Fisher Scientific, Whitby, ON) before reaching the O_2_ and CO_2_ analyzers (Qubit Systems Inc., Kingston, ON). Reference gas calibration was performed using standard gas mixtures (nitrogen and carbon dioxide) at two concentrations (99.98% and 1.01%), once in the morning and once in the afternoon after the dogs were placed back in the chambers from being outside. The first measurement after the dogs returned from outside was always discarded. Respiratory gases were measured for 5 min every 25 min. Measurements took place as follows: fasted (1.5 h prior to meal), postprandial (0 to 4 h post-meal) and post-absorptive (5 to 10 h post-meal). Dogs were fed 100% of their daily ration after 3 fasted measures were completed. If the dogs’ daily ME intake were adjusted in order to maintain BW, they were fed the same amount, on an energy basis, as the first measurement on each consecutive calorimetry day for consistency among treatments and because energy intake is thermogenic. Finally, RQ was calculated within the C950 Multi Channel Gas Exchange Software (Qubit Systems Inc.) and EE was calculated from O_2_ consumption and CO_2_ production using the abbreviated Weir (1949) equation and expressed on a per kg metabolic BW basis (BW^0.75^).


$$\begin{array}{l} EE\;\left(\frac{kcal}{h}\right)=\left(3.94\;\times \;VO_{2}\right)+\left(1.1\;\times \;VCO_{2}\right) \end{array}$$

Where $VO_{2}$ is the volume of oxygen consumed (L/min) and $VCO_{2}$ is the volume of carbon dioxide produced (L/min).

### Serum Total Lipid Analysis

Lipid extraction was done according to Folch et al. (1957). Serum samples were thawed on ice before 50 μL of serum was added to 950 μL 0.1 M KCl (Sigma-Aldrich, Oakville, ON). Four mL of 2:1 CHCl_3_: MeOH (Sigma-Aldrich) containing 17:0 standard was added and the samples were vortexed, flushed with nitrogen, and kept overnight at 4 °C. Samples were centrifuged at 1460 rpm for 10 min at 21 °C and the chloroform layer was collected and dried down under nitrogen. Two mL of 0.5 M KOH (Sigma-Aldrich) in methanol was added to each sample and saponified at 100 °C for 1 h. 2 mL hexane and 2 mL 14% BF_3_-MeOH (Sigma-Aldrich) was added to each sample and then methylated at 100 °C for 1 h. 2 mL of double distilled water was added to stop methylation and samples were centrifuged at 1460 rpm for 10 min at 21 °C. The top hexane layer was removed and dried down under nitrogen. Samples were reconstituted in 100 μL of hexane for analysis.

Fatty acid methyl esters were quantified on an Agilent 6890 gas chromatograph equipped with flame ionization detection and separated on an DB-FFAP fused-silica capillary column (15 m, 0.1 μm film thickness, 0.1 mm i.d.; Agilent Cat# 127-32H2). Samples were injected in 200:1 split mode. The injector and detector ports were set at 250 °C. Fatty acid methyl esters were eluted using a temperature program set initially at 150 °C and held for 0.25 min, increased at 35 °C /min and held at 170 °C for 3 min, increased at 9 °C /min to 225 °C, and finally increased 80 °C/min to 245 °C and held for 2.2 min. The run time per sample was 12 min. The carrier gas was hydrogen, set to a 30 mL/min constant flow rate. Peaks were identified by retention times of fatty acid methyl ester standards (Nu-Chek-Prep, Elysian, MN) using EZchrom Elite version 3.2.1 software. Fatty acid concentrations were calculated as percent area.

### Statistical Analysis

Body weight, food intake, caloric intake, macronutrient intake, serum BOHB and serum free fatty acids were analyzed as repeated measures using the proc glimmix function in SAS Studio (v 9.4; SAS Institute Inc., Cary, NC) with dog and period treated as random effects. Serum BOHB and free fatty acid concentrations at week -1 were used as covariates in their respective models. The effect of treatment, week and treatment × week were evaluated. Serum biochemistry and CBC were analyzed using the proc glimmix function in SAS Studio with dog and period treated as random effects. Biochemistry values at week -1 was used as a covariate. Serum bilirubin was analyzed assuming a multinomial distribution and the cumulative logit link function was used, given that reported values were discrete and not continuous. The effect of treatment was evaluated.

Respiratory quotient and EE corrected for metabolic body weight was analyzed at fasted, 0 to 4 h post-meal and 5 to 10 h post-meal. Data was analyzed as doubly repeated measures using the mixed function in SAS Studio where week and time were repeated with dog nested within treatment as the subject. In the statistical model, the effect of treatment, time, week, treatment × time interaction and treatment × week interaction were evaluated. The three-way interaction between treatment, time post-meal and week was not evaluated due to lack of biological relevance related to the objectives of the study. The direct product compound symmetry (UN@CS) covariance structure (appropriate for multivariate repeated measures) was used. For all outcomes, model assumptions were assessed through residual analysis and if assumptions were violated, a log-transformation was performed. Means were separated using the Tukey-Kramer adjustment and significance was declared at P ≤ 0.05.

## RESULTS

### Body Weight and Food Intake

Despite feeding to maintain BW, there were significant treatment × time interaction effects for BW (kg), food intake (g/day), and caloric intake (kcal/day; P < 0.05, Table 3). Briefly, BW was greater in dogs fed HPLC than dogs fed MET at weeks 5 and 6 but MPMC was similar to both, despite food intake being greatest in dogs fed MET across all weeks. Calculated ME intake, according to the Modified Atwater equation, as required by the Association of American Feed Control Officials (AAFCO; Beckman, 2023) was greater in dogs fed MET than dogs fed MPMC at week 5 and greater than both MPMC and HPLC at week 6. Calculated ME intake, according to the National Research Council (NRC, 2006) using crude fiber (CF) was greater in dogs fed HPLC and MPMC than dogs fed MET at week 1 and greater in dogs fed HPLC compared to dogs fed MET at weeks 2, 3 and 4. Calculated ME intake according to the NRC (2006) using TDF was greater in dogs fed HPLC compared to dogs fed MET at weeks 1 and 2 but greater in dogs fed MET compared to dogs fed MPMC at week 6. In addition, dogs fed HPLC had a greater crude protein (CP) and fat intake (g/day) than dogs fed MPMC and MET across all weeks (P < 0.05) and a lower NFE intake than dogs fed MPMC and MET across all weeks (P < 0.05). Dogs fed MET had a greater TDF intake compared to dogs fed HPLC and MPMC across all weeks (P < 0.05).

**Table 3.** Effect of diet [high protein, low carbohydrate (HPLC), moderate protein, moderate carbohydrate (MPMC) and metabolic (MET)] on body weight, food intake and calorie intake of dogs (n = 9) over 6 wk

Trt	Week						SEM	P-value		
	1	2	3	4	5	6		Trt	Week	Trt × Week
Body weight, kg										
HPLC	26.4	26.6	26.7	26.7	26.9^a^	26.9^a^	0.8	0.058	0.082	**0.023**
MPMC	26.4	26.5	26.6	26.6	26.7^ab^	26.6^ab^				
MET	26.6	26.5	26.5	26.4	26.4^b^	26.3^b^				
Food intake, g/d										
HPLC	404.5^b^	398.3^b^	392.7^b^	392.8^b^	378.8^b^	362.3^b^	25.2	**<0.001**	0.223	**<0.001**
MPMC	407.7^b^	401.4^b^	396.8^b^	388.9^b^	381.5^b^	377.2^b^				
MET	449.5^a^	451.7^a^	454.1^a^	460.8^a^	475.1^a^	482.2^a^				
Calorie intake (ME_AAFCO_), kcal/d										
HPLC	1541.6	1518.2	1496.6	1497.2	1443.8^ab^	1380.9^b^	82.2	0.412	0.101	**<0.001**
MPMC	1482.8	1460.0	1443.2	1414.7	1387.8^b^	1372.0^b^				
MET	1442.8	1449.9	1457.7	1479.4	1525.1^a^	1547.8^a^				
Calorie intake (ME_NRC_CF_), kcal/d										
HPLC	1636.4^a^	1611.6^a^	1588.7^a^	1589.3^a^	1532.7	1465.9	83.9	**0.045**	0.064	**<0.001**
MPMC	1557.6^a^	1533.6^ab^	1516.0^ab^	1486.1^ab^	1457.7	1441.2				
MET	1405.5^b^	1412.4^b^	1420.1^b^	1441.1^b^	1485.6	1505.8				
Calorie intake (ME_NRC_TDF_), kcal/d										
HPLC	1707.1^a^	1681.2^a^	1657.3	1658.0	1598.9	1529.3^ab^	89.0	0.213	0.084	**<0.001**
MPMC	1608.6^ab^	1583.9^ab^	1565.6	1534.8	1505.5	1488.4^b^				
MET	1535.4^b^	1542.9^b^	1551.3	1574.3	1622.9	1647.1^a^				
Crude Protein Intake (g/day)										
HPLC	210.7^a^	207.5^a^	204.6^a^	204.7^a^	197.4^a^	188.8^a^	8.4	**<0.001**	**0.024**	**<0.001**
MPMC	117^b^	115.2^b^	113.9^b^	111.6^b^	109.5^c^	108.3^c^				
MET	122.3^b^	122.9^b^	123.5^b^	125.4^b^	129.2^b^	131.2^b^				
Crude Fat Intake (g/day)										
HPLC	76.9^a^	75.7^a^	74.6^a^	74.6^a^	72.0^a^	68.8^a^	3.5	**<0.001**	**0.041**	**<0.001**
MPMC	59.1^b^	58.2^b^	57.5^b^	56.4^b^	55.3^b^	54.7^b^				
MET	56.2^b^	56.5^b^	56.8^b^	57.6^b^	59.4^b^	60.3^b^				
Total Dietary Fiber Intake (g/day)										
HPLC	28.3^b^	27.9^b^	27.5^b^	27.5^b^	26.5^b^	25.4^b^	2.9	**<0.001**	0.9170	**<0.001**
MPMC	32.2^b^	31.7^b^	31.3^b^	30.7^b^	30.1^b^	29.8^b^				
MET	71.9^a^	72.3^a^	72.7^a^	73.7^a^	76.0^a^	77.1^a^				
NFE Intake (g/day)										
HPLC	39.6^b^	39.0^c^	38.5^c^	38.5^c^	37.1^c^	35.5^c^	7.2	**<0.001**	0.911	**<0.001**
MPMC	157.0^a^	154.5^b^	152.8^b^	149.7^b^	146.9^b^	145.2^b^				
MET	167.7^a^	168.5^a^	169.4^a^	171.9^a^	177.2^a^	179.8^a^				

^a,b,c^Different letters within the same column indicate statistical significance (P < 0.05).

### Serum Biochemistry and Complete Blood Count

All parameters measured on the complete blood count were similar among the three treatment groups and remained within reference range (P > 0.05, Supplementary Table 1).

Several biochemistry parameters differed among diets (Table 4). Despite the null hypothesis being rejected in the F-test (P = 0.031), no significant differences (P > 0.05) were observed among diets for phosphorus when pairwise comparisons were analyzed using the Tukey–Kramer adjustment. Serum urea concentrations were greater in dogs fed HPLC than dogs fed MPMC or MET (P < 0.001). Serum creatinine concentrations were greatest in MPMC, followed by HPLC and then MET (P < 0.001). Finally, serum alkaline phosphatase was greater in dogs fed MET than dogs fed HPLC, and MPMC was similar to both (P = 0.025). Despite these differences, all parameters remained within standard reference ranges. Serum conjugated, free and total bilirubin were not different between treatments (P > 0.05, data not shown).

**Table 4.** Effect of diet on serum biochemistry parameters in dogs (n = 9) at the end of 6 wk of feeding the high protein, low carbohydrate (HPLC), moderate protein, moderate carbohydrate (MPMC) and metabolic (MET) diet

Parameter, unit	Trt			SEM	P-value	Reference interval^1^
	HPLC	MPMC	MET			
Calcium, mmol/L	2.46	2.51	2.50	0.053	0.118	2.50–3.00
Phosphorus, mmol/L	1.12	1.19	1.12	0.042	**0.031**	0.90–1.85
Magnesium, mmol/L	0.8	0.8	0.8	0.015	0.187	0.7–1.0
Sodium, mmol/L	144	144	144	1.100	0.837	140–154
Potassium, mmol/L	4.5	4.5	4.5	0.075	0.888	3.8–5.4
Chloride, mmol/L	111	111	111	1.065	0.607	104–119
Carbon dioxide, mmol/L	19	19	20	0.447	0.245	15–25
Anion gap, mmol/L	18	19	18	0.391	0.246	13–24
Na:K ratio	32	32	32	0.430	0.687	29–37
Total Protein, g/L	61	62	61	0.588	0.785	55–74
Albumin, g/L	38	39	38	0.578	0.345	29–43
Globulin, g/L	23	23	23	0.861	0.704	21–42
Albumin:Globulin ratio	1.6	1.7	1.6	0.082	0.358	0.7–1.8
Urea, mmol/L	6.5^a^	5.7^b^	5.3^b^	0.187	**<0.001**	3.5–9.0
Creatinine, µmol/L	73^b^	86^a^	66^c^	1.491	**<0.001**	20–150
Glucose, mmol/L	5.2	5.3	5.0	0.094	0.071	3.3–7.3
Cholesterol, mmol/L	6.27	6.30	6.39	0.221	0.932	3.60–10.20
High density lipoprotein, mmol/L	5.3	5.2	5.4	0.129	0.500	
Non-high density lipoprotein, mmol/L, calculated^2^	1.03	1.12	0.94	0.137	0.377	
Alkaline phosphatase, U/L	42^b^	55^ab^	75^a^	7.934	**0.025**	22–143
Steroid induced ALP, U/L	18	21	21	3.576	0.402	0–84
Gamma-glutamyltransferase, U/L	3	3	3	0.536	0.352	0–7
Alanine aminotransferase, U/L	36	26	39	6.563	0.353	19–107
Creatinine kinase, U/L	100	137	99	26.517	0.470	40–255
Amylase, U/L	629	739	646	46.463	0.208	299–947
Lipase, U/L	127	136	100	20.582	0.444	25–353
Calculated osmolarity, mmol/L	288	288	287	2.323	0.769	

^1^Animal Health Laboratory, University of Guelph, ON. Values established using 86 clinically healthy, adult dogs of various breeds.

^2^Non-high density lipoprotein = cholesterol—high density lipoprotein.

^a,b,c^Different letters within the same row indicate statistical significance (P < 0.05).

### β-hydroxybutyrate

Serum BOHB concentrations were greater on week 6 than week 2 but both were similar to week 4 (P = 0.010; Table 5). There were no differences among treatments or treatment over week (P > 0.05).

**Table 5.** Effect of diet [high protein, low carbohydrate (HPLC), moderate protein, moderate carbohydrate (MPMC) and metabolic (MET) diet] on the β-hydroxybutyrate concentration (µmol/L) in dogs (n = 9) over 6 wk

	Trt				Week				P-value			
	HPLC	MPMC	MET	SEM	2	4	6	SEM	Trt	Week	Trt × Week	Reference Interval^1^
β-hydroxybutyrate concentration (µmol/L)	15	11	11	3	8^b^	12^ab^	18^a^	3	0.274	**0.010**	0.432	0–200

^1^Animal Health Laboratory, University of Guelph, ON. Values established using 86 clinically healthy, adult dogs of various breeds.

^a,b,c^Different letters within the same row indicate statistical significance (P < 0.05).

### Indirect Calorimetry

**Figure 1.**
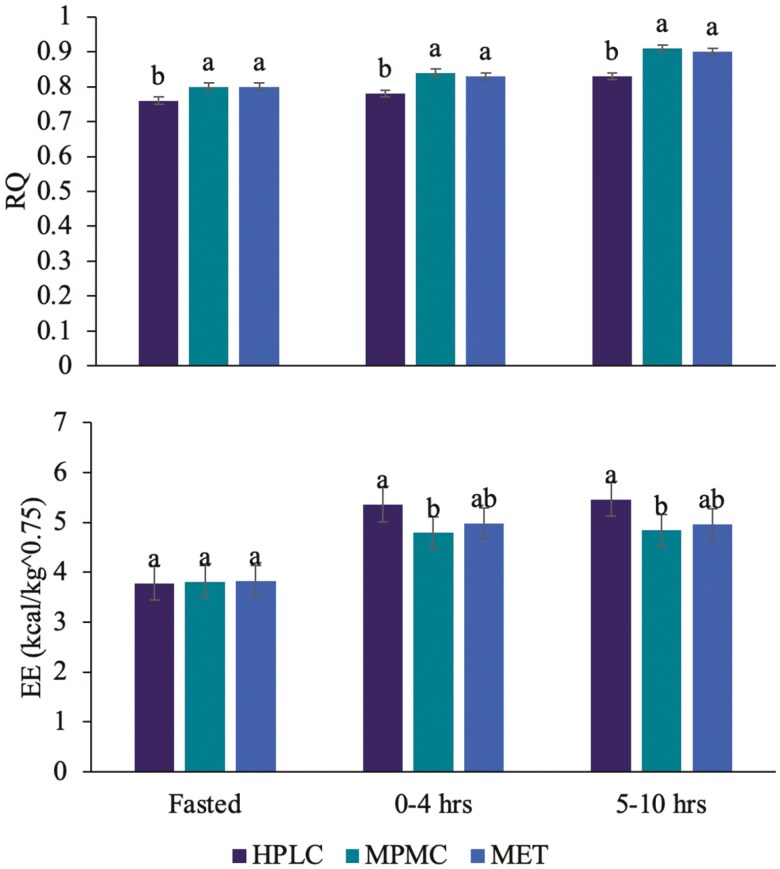
Respiratory quotient (RQ, SEM = 0.9) and energy expenditure (EE, SEM = 5.5) among dogs fed the high protein, low carbohydrate (HPLC), moderate protein, moderate carbohydrate (MPMC) and metabolic (MET) diet at fasted, postprandial (0 to 4 h post-meal) and postabsorptive (5 to 10 h post-meal) across weeks (n = 8). Different letters within the same time point indicate significance at P < 0.05.

Both RQ (P = 0.002) and EE corrected for metabolic BW (P = 0.034) had a significant treatment × time interaction effect (Figure 1), but were not different across weeks or treatment × week. Dogs fed HPLC had a significantly lower RQ at fasted, 0 to 4 h and 5 to 10 h post-meal compared to dogs fed MET and MPMC. All dogs had a similar EE at fasted but at 0 to 4 h and 5 to 10 h post-meal, dogs fed HPLC had a significantly greater EE than dogs fed MPMC but dogs fed MET were similar to both.

### Serum Total Lipids

**Figure 2.**
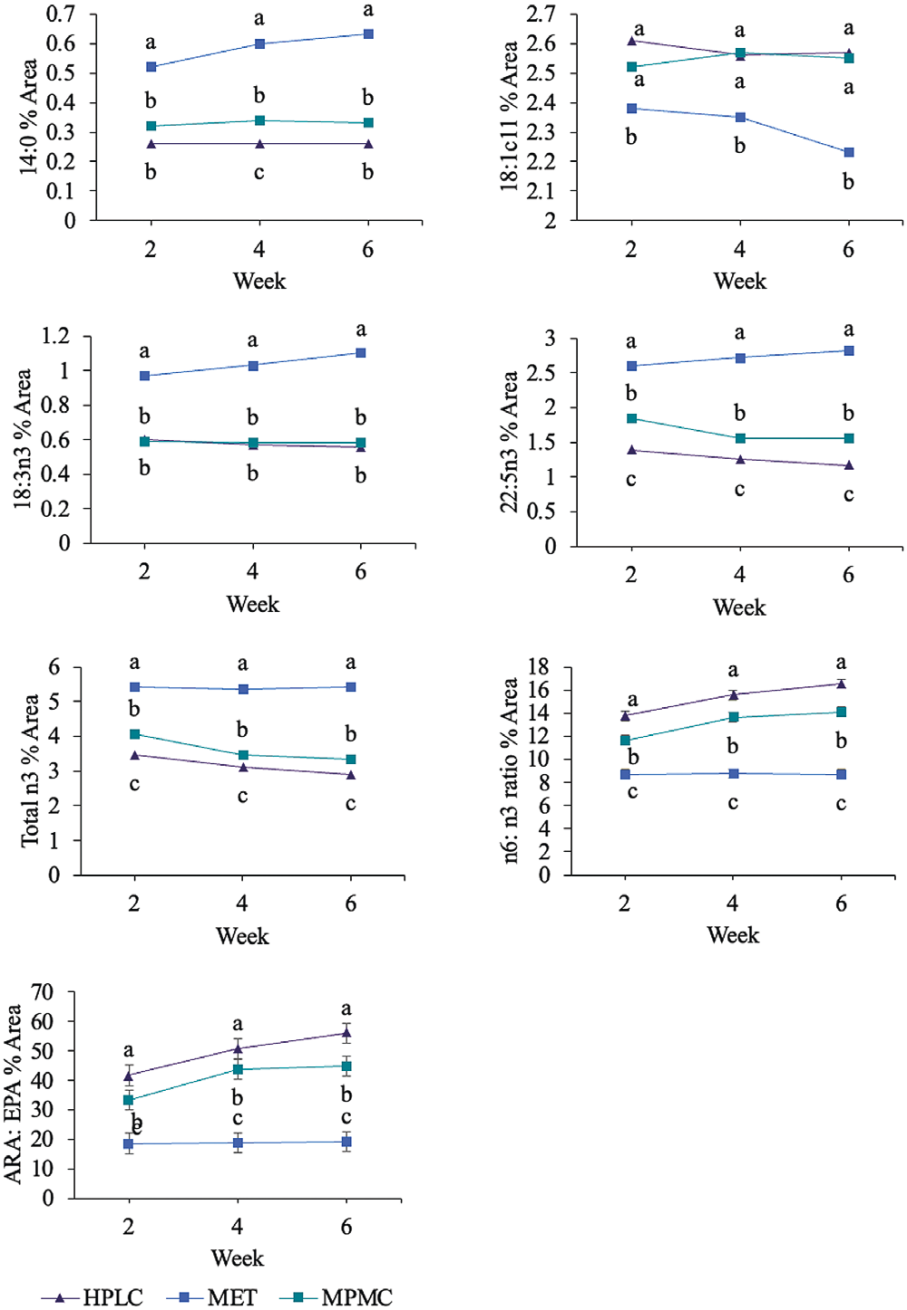
Fatty acids with a significant treatment by week effect in dogs (n = 9) fed the high protein, low carbohydrate (HPLC), moderate protein, moderate carbohydrate (MPMC), and metabolic (MET) diet over 6 weeks. Different letters indicate statistical significance at P < 0.05. Myristic acid (14:0, top left, SEM = 0.02), cis-vaccenic acid (18:1c11, top right, SEM = 0.04), alpha-linolenic acid (18:3n3, top middle left, SEM = 0.04), n-3 docosapentaenoic acid (22:5n3, top middle right, SEM = 0.08), total n3 (bottom middle left, SEM = 0.09), total n6:n3 ratio (bottom middle right, SEM = 0.4), and total arachidonic acid (ARA): eicosapentaenoic acid (EPA; 20:4n6:20:5n3, bottom left, SEM = 3.4).

The majority of fatty acids had a significant treatment effect (Table 6). Five fatty acids and two ratios had a significant treatment × week interaction effect (Figure 2). Myristic acid (14:0), alpha-linolenic acid (ALA; 18:3n3), n3 docosapentaenoic acid (DPA; 22:5n3) and total n3 were greatest in dogs fed MET at each week (P < 0.05). In contrast, cis-vaccenic acid (18:1c11), total n6: n3 and ARA (20:4n6): EPA (20:5n3) were all greater in dogs fed HPLC and MPMC compared to dogs fed MET at each week (P < 0.05).

**Table 6.** Serum free fatty acids (% area) across treatment [high protein, low carbohydrate (HPLC), moderate protein, moderate carbohydrate (MPMC) and metabolic (MET)] and week in all dogs (n = 9)

	Trt				Week				P-value		
Fatty acid^1^	HPLC	MPMC	MET	SEM	2	4	6	SEM	Trt	Week	Trt × Week
Lauric acid (12:0)	0.06^b^	0.05^b^	0.10^a^	0.007	0.04^b^	0.08^a^	0.08^a^	0.007	**0.002**	**<0.001**	0.335
Myristic acid (14:0)	0.26	0.33	0.58	0.02	0.36	0.40	0.41	0.01	**<0.001**	**0.003**	**0.007**
Pentadecylic acid (15:0)	0.123^b^	0.136^ab^	0.140^a^	0.006	0.13	0.13	0.14	0.004	**0.003**	0.133	0.610
Palmitic acid (16:0)	14.65^ab^	14.98^a^	14.30^b^	0.15	14.46^b^	14.73^a^	14.74^a^	0.14	**0.001**	0.350	0.612
Stearic acid (18:0)	16.61^ab^	16.36^b^	17.23^a^	0.23	16.68	16.73	16.77	0.15	**0.034**	0.842	0.535
Nonadecanoic acid (19:0)	0.17^b^	0.21^a^	0.23^a^	0.01	0.19^b^	0.21^a^	0.20^ab^	0.01	**<0.001**	0.051	0.967
Arachidic acid (20:0)	0.15^b^	0.17^a^	0.17^a^	0.01	0.16	0.16	0.17	0.01	**0.020**	0.271	0.224
Behenic acid (22:0)	0.77^a^	0.68^ab^	0.65^b^	0.12	0.78^a^	0.66^b^	0.66^b^	0.12	**0.012**	**<0.001**	0.074
Tricosanoic acid (23:0)	0.17^ab^	0.17^b^	0.19^a^	0.007	0.20^a^	0.17^b^	0.16^b^	0.007	**0.019**	**<0.001**	0.992
Lignoceric acid (24:0)	0.23	0.25	0.26	0.02	0.24	0.25	0.24	0.02	0.058	0.420	0.703
Total SFAs	33.17	33.30	33.87	0.24	33.25	33.52	33.56	0.20	0.097	0.365	0.474
Palmitoleic acid (16:1c9)	1.31^b^	1.27^a^	0.92^a^	0.05	1.14	1.17	1.19	0.04	**<0.001**	0.376	0.198
Oleic acid (18:1c9)	10.54^a^	10.87^a^	9.12^b^	0.19	10.25	10.14	10.15	0.13	**<0.001**	0.599	0.451
Gondoic acid (18:1c11)	2.58	2.54	2.32	0.03	2.50	2.49	2.45	0.03	**<0.001**	0.079	**0.013**
Gondoic acid (20:1c11)	0.21	0.23	0.21	0.01	0.24^a^	0.21^b^	0.21^b^	0.01	0.222	**0.004**	0.701
Mead acid (20:3n9)	0.20^b^	0.25^a^	0.17^b^	0.01	0.218^a^	0.203^ab^	0.202^b^	0.009	**<0.001**	**0.027**	0.351
Erucic acid (22:1n9)	0.46	0.42	0.40	0.04	0.40	0.45	0.42	0.04	0.187	0.375	0.627
Nervonic acid (24:1n9)	0.50^a^	0.45^ab^	0.43^b^	0.02	0.45	0.47	0.46	0.02	**0.034**	0.305	0.719
Total MUFA	15.80^a^	16.01^a^	13.61^b^	0.26	15.20	15.14	15.08	0.18	**<0.001**	0.848	0.593
Linoleic acid (18:2n6)	23.18^c^	25.13^b^	27.19^a^	0.41	25.17	25.09	25.24	0.35	**<0.001**	0.935	0.800
Gamma-Linolenic acid (18:3n6)	0.41	0.39	0.37	0.02	0.38	0.40	0.39	0.01	0.194	0.070	0.387
Alpha-linolenic acid (18:3n3)	0.57	0.58	1.03	0.03	0.72	0.72	0.74	0.03	**<0.001**	0.385	**0.010**
Stearidonic acid (18:4n3)	0.13	0.13	0.15	0.007	0.14	0.14	0.14	0.006	0.195	0.997	0.521
Eicosadienoic acid (20:2n6)	0.20^c^	0.26^b^	0.29^a^	0.009	0.26	0.24	0.25	0.007	**<0.001**	0.242	0.492
Dihomo-gamma linolenic acid (20:3n6)	0.88^ab^	0.92^a^	0.82^b^	0.02	0.88^ab^	0.86^b^	0.89^a^	0.02	**0.018**	**0.008**	0.163
Arachidonic acid (20:4n6)	21.10^a^	18.59^b^	17.03^c^	0.43	18.89	18.97	18.82	0.33	**<0.001**	0.699	0.904
Eicosatrienoic acid (20:3n3)	0.04^b^	0.03^b^	0.08^a^	0.02	0.04	0.06	0.05	0.02	**<0.001**	0.088	0.930
Eicosapentaenoic acid (20:5n3)	0.45^b^	0.49^b^	0.89^a^	0.03	0.67^a^	0.59^b^	0.56^b^	0.02	**<0.001**	**<0.001**	0.107
Adrenic acid (22:4n6)	1.51^a^	1.50^a^	1.24^b^	0.06	1.33^c^	1.42^b^	1.50^a^	0.04	**0.004**	**<0.001**	0.350
n-6 Docosapentaenoic acid (22:5n6)	0.37^a^	0.37^a^	0.28^b^	0.02	0.30^b^	0.35^a^	0.37^a^	0.01	**0.009**	**0.001**	0.653
n-3 Docosapentaenoic acid (22:5n3)	1.27	1.65	2.71	0.07	1.94	1.84	1.85	0.06	**<0.001**	**0.006**	**<0.001**
Docosahexaenoic acid (22:6n3)	0.65^ab^	0.76^a^	0.60^b^	0.03	0.81^a^	0.63^b^	0.56^c^	0.02	**0.004**	**<0.001**	0.087
20:4n6: 22:5n3	49.57	40.60	18.84	3.12	31.20	37.78	40.03	2.81	**<0.001**	**<0.001**	**0.001**
Total PUFA	51.52^b^	51.32^b^	53.58^a^	0.36	52.26	52.06	52.09	0.34	**<0.001**	0.798	0.463
Total n6	47.77	47.09	47.16	0.35	47.23	47.35	47.44	0.32	0.272	0.852	0.776
Total n3	3.17	3.62	5.41	0.08	4.32	3.98	3.90	0.05	**<0.001**	**<0.001**	**<0.001**
n6:n3	15.29	13.13	8.72	0.34	11.37	12.66	13.12	0.30	**<0.001**	**<0.001**	**0.002**

^1^Fatty acids with <0.01% area in all samples were removed from the table.

^a,b,c^Different letters within the same row indicate statistical significance (P < 0.05).Abbreviations: saturated fatty acids (SFAs), monounsaturated fatty acids (MUFAs), polyunsaturated fatty acids (PUFAs), total n6 fatty acids, total n3 fatty acids, n6:n3 ratio.

Although most saturated fatty acids (SFAs) were different among treatments, the total SFAs were not different among treatments (P > 0.05). The total monounsaturated fatty acids (MUFAs) were greater in dogs fed HPLC and MPMC compared to dogs fed MET (P < 0.001). In contrast, the total polyunsaturated fatty acids (PUFAs) were greater in dogs fed MET compared to dogs fed HPLC and MPMC (P < 0.001). Although there was no difference in total n6 fatty acids among diets, dogs fed MET had greater total n3 fatty acids compared to dogs fed HPLC and MPMC (P < 0.001), leading to a lower n6: n3 in dogs fed MET as well (8.72; P < 0.001). In terms of concentrations over week, lauric acid (12:0), adrenic acid (22:4n6), dihomo-gamma linolenic acid (20:3n6) and n6 DPA (22:5n6) increased over week (P < 0.05), whereas behenic acid (22:0), tricosanoic acid (23:0), gondoic acid (20:1c11), mead acid (20:3n9), EPA (20:5n3), and docosahexaenoic acid (DHA; 22:6n3) decreased over week (P < 0.05).

## DISCUSSION

In partial support of our hypothesis, dogs fed HPLC had a lower postprandial RQ than dogs fed MPMC and MET and a greater EE compared to dogs fed MPMC but not different from dogs fed MET. This suggests greater fatty acid oxidation for EE. Interestingly, this was also maintained at fasting for RQ, but not for EE. This was an expected outcome of feeding a HPLC diet and one reason why these diets are often used for weight loss in humans (Moon and Koh, 2020). In dogs, the feeding of a HPLC diet to overweight dogs resulted in greater weight loss when the diets were restricted to 85% of the dog’s energy requirements compared to a control MPMC diet (Bierer and Bui, 2004). The dogs in the present study were lean and despite a higher EE and lower RQ, dogs fed HPLC had a higher BW and lower calculated ME intake [according to the Modified Atwater equation as required by AAFCO (Beckman, 2023)] than dogs fed MET by week 6. However, when energy density of the diet was calculated using the equations provided by the NRC (2006), the opposite trend was observed where dogs fed HPLC consumed more calories in the first weeks of the study than dogs fed MET but similar calories by weeks 5 and 6. It is widely accepted that the NRC formulas are more accurate because the proposed macronutrient gross energy coefficients were determined prior to subtraction of estimated urinary losses (Traughber et al., 2021). In addition, gross energy can be determined using bomb calorimetry and different calculations are provided for either CF or TDF (Traughber et al., 2021). Crude fiber is known to underestimate fiber content compared to TDF, leading to a lower calculated ME and potential overfeeding (Traughber et al., 2021). Therefore, it is likely that the ME of the HPLC diet was overestimated to a greater extent and the dogs were overfed in the first few weeks of the study, given that the Modified Atwater equation was used to calculate the ME of the diets to be fed, which led to an increase in BW in dogs fed HPLC by week 6. However, the greater total dietary fiber intake of the MET diet, along with the L-carnitine supplementation is important to consider given that L-carnitine has been demonstrated to increase EE in exercising dogs (Varney et al., 2020) as well as in overweight cats fed at maintenance (Shoveller et al., 2014). In addition, diets with TDF concentrations >12% are known to be less digestible (Fahey et al., 1990). This may help to explain why dogs fed MET had a lower BW by week 6 and the postprandial EE of dogs fed MET was not different from dogs fed HPLC. In addition, Shoveller et al. (2014) reported no difference in EE in lean cats fed at maintenance and supplemented with L-carnitine, which also helps explain why the EE of dogs fed MET was not different from dogs fed MPMC, as these were lean dogs.

Data on the length of time required for metabolic adaptation to a low carbohydrate diet is variable. One study in human athletes reports adaptation to changes in nutrient oxidation during exercise in as little as 5 to 6 d of consuming a low carbohydrate diet (Burke et al., 2021). It appears to take longer for EE adaptation to a low carbohydrate diet in humans where a meta-analysis of studies lasting from 1 to 140 d and carbohydrate contents of 8% to 77% concluded that total EE only slightly decreased in studies less than 2.5 wk but substantially decreased in studies longer than 2.5 wk (Ludwig et al., 2021). The present study found no difference between EE or RQ between the measured timepoints at week 3 and week 6, aligning with this finding of a 2.5 wk adaptation period for EE. If it took longer than 3 wk for adaptation, we would expect differences in EE between our measurements at 3 versus 6 wk. However, this may depend on the specific outcome of interest as several of the fatty acids that we measured changed over the 6 wk, including ALA and DPA.

It is expected that the serum fatty acid profile of dogs should mirror the dietary fatty acid profile (Table 1). This was generally the case for individual fatty acids and MUFAs, as dogs fed MET had the lowest serum total MUFAs and lowest dietary total MUFAs. However, dogs fed MET had the highest serum total PUFAs but the lowest dietary total PUFAs. This is likely due to two factors. First, two fatty acids that were not measured in the diet, eicosatrienoic acid and ecosapentaenoic acid, were measured in serum and were greater in dogs fed MET. Second, dietary linoleic acid (LA) was lower in the MET diet, but highest in the serum of dogs consuming MET compared to MPMC and HPLC. Given that LA cannot be synthesized by mammals and ALA and LA compete for the same desaturase enzymes (Calder, 2011), perhaps the greater concentrations of ALA in the MET diet prevented the desaturation and elongation of LA by these enzymes to allow more LA accumulation in the dogs fed MET. Interestingly, while all three diets had negligible amounts of DPA, DHA and EPA (not shown Table 1) due to no marine sources being added to any diet, EPA and DPA were greater in dogs fed MET compared to dogs fed HPLC and MPMC and DHA was greater in dogs fed MPMC compared to dogs fed MET but HPLC was similar to both. Typically, the conversion of ALA to EPA, DHA and DPA is low in mammals (<10% in humans and likely similar in dogs; Dunbar and Bauer, 2002), however, because MET had higher levels of ALA and lower levels of LA (thus a lower LA: ALA) in the diet, it may have led to a relatively larger conversion to EPA and DPA than the HPLC and MPMC diets due to the competition for enzymes between the LA versus ALA pathways. This suggests a potential role for ALA in improving overall n3 status (Burron et al., 2024).

Also as hypothesized, dogs fed HPLC had a greater n6: n3 and a greater ARA: EPA than dogs fed MET and MPMC, aligning with previous literature in dogs fed a HPLC diet (Jackson, 2022) and humans fed very low carbohydrate diets (<12%; Johnston et al., 2006; Forsythe et al., 2008). This somewhat aligns with dietary levels as the HPLC diet had the greatest concentration of ARA and total n6. In general, n6’s are more involved in pro-inflammatory processes, especially ARA, as it gives rise to pro-inflammatory eicosanoids, whereas n3’s are more involved in anti-inflammatory processes, especially EPA and DHA, which give rise to anti-inflammatory resolvins (Calder, 2011). However, Forsythe et al. (2008) reported lower concentrations of some pro-inflammatory cytokines, chemokines and adhesion molecules in overweight people consuming a very low carbohydrate diet (12%) compared to a low fat diet (24%), despite higher ARA concentrations. The authors argue that the higher ARA suggests a lower conversion rate to pro-inflammatory eicosanoids because there was no increase in ARA precursors (ALA and dihomo-gamma linolenic acid), so increased synthesis of ARA from LA was unlikely. Furthermore, the dietary contribution of ARA to the total pool is very small. Although we did not measure cytokines in the present study, it is often reported that low carbohydrate diets can decrease inflammation via lower pro-inflammatory cytokine concentrations in humans (Sharman and Volek, 2004; Jonasson et al., 2014; Kazeminasab et al., 2024). However, this may be due to the weight loss induced by these diets rather than the diets themselves (Sharman and Volek, 2004). As these dogs were lean, fed to maintain BW, and did not experience any immune challenge, it is unlikely we would have seen differences in inflammatory cytokines had we measured them. However, Tavener et al. (2024) reported lower pro-inflammatory cytokines and chemokines and greater ARA concentrations in healthy Beagles fed a HPLC (53% of ME from protein, 8% of ME from carbohydrate) and a high fat (69% of ME), low carbohydrate (5% of ME) diet compared to a control MPMC diet after 5 wk. Interestingly, these dogs needed to consume more calories on the HPLC and high fat, low carbohydrate diets than the MPMC diet in order to maintain weight, contrary to the present study. Plasma and tissue ARA decreases in early obesity-induced insulin resistant mice (Fukuchi et al., 2004) and supplemental ARA lowers glucose concentrations after an oral glucose tolerance test in overweight mice (Wu et al., 2007). This is interesting because HPLC diets are often used in the treatment of type 2 diabetes for their reported ability to lower glucose and increase insulin sensitivity (Seino et al., 1983; Gannon and Nuttall, 2004), suggesting a potential role for the elevated ARA in the blood of individuals fed HPLC diets in the prevention of type 2 diabetes (Das, 2018). This is thought to occur through the Cytochrome P450—soluble epoxide hydrolase pathway, where PUFA-epoxides can be produced from ARA and act as bioactive compounds. This is in contrast to the eicosanoids produced by ARA that are pro-inflammatory as ARA-epoxides can play a role in both insulin-secreting and anti-inflammatory pathways (Isse et al., 2022). However, the data is complex and the exact mechanism by which these ARA-epoxides may help prevent type 2 diabetes is currently unclear. Indeed, further research focused on how diet affects fatty acid metabolism in different physiological states is needed to further advance this field.

In contrast to several other studies that report greater HDL in both overweight humans (Sharman et al., 2004; Forsythe et al., 2008) and healthy weight humans (Sharman et al., 2002; Volek et al., 2003) consuming very low carbohydrate (<12%), high protein (>28%), high fat (>59%) diets, we found no difference in total or HDL cholesterol among any diets. The studies in healthy adults above report favorable increases in HDL in as little as 4 wk. Our study used healthy dogs fed HPLC for 6 wk, so it is likely that the ratio of carbohydrate to fat was higher than that of the very low carbohydrate diets used in the human studies. Although the HPLC diet did have a very low calculated NFE content (~11%), the fat content was not nearly as high as those in the human studies above. When determining if a diet is considered “ketogenic,” it is the fat content that plays a larger role, and the ketogenic ratio [KR = (0.9 × g of fat) + (0.46 × g of protein) / g of carbohydrate + (0.1 × g of fat) + (0.58 × g of protein)] must be 1.5 or greater (Zilberter and Zilberter, 2018). The HPLC diet had a ketogenic ratio of 0.96, compared to the other two diets being ~0.45. This, along with the high variation in analysis and reported SEM, may also explain why the BOHB concentrations were not different among diets. In a study done in healthy adult Beagles, BOHB concentrations were only greater in dogs fed a high fat (69% of ME), low carbohydrate (5% of ME, KR = 1.63) diet, but not a HPLC (53% of ME from protein, 8% of ME from carbohydrate, KR = 0.97) diet compared to the control MPMC diet after 5 wk (Jackson, 2022), similar to the present study. It is also possible that we did not see differences in total or HDL cholesterol because dogs are considered to be HDL-dominant mammals in contrast to humans which are LDL-dominant mammals, therefore their cholesterol metabolism is quite different from humans (Mori et al., 2011). However, in the same study as above, dogs fed the high fat, low carbohydrate diet had greater total cholesterol than dogs fed the HPLC diet who had greater total cholesterol than dogs fed the control MPMC diet (Jackson, 2022). However, these authors did not measure HDL cholesterol making further interpretation difficult.

Finally, all health parameters measured on the complete blood count and biochemistry analysis remained within the healthy reference range, suggesting no adverse effects of consuming a HPLC diet for 6 wk. Serum urea and creatinine can be affected by dietary protein intake (Watson et al., 1981). As expected, urea was higher in dogs fed HPLC than dogs fed MET or MPMC due to the higher protein intake. However, creatinine was greater in dogs fed MPMC than dogs fed HPLC. Few external factors effect serum creatinine, the main ones being diet and muscle mass (Braun et al., 2003). Given these were all the same breed and size dogs and each consumed all diets, differences in muscle mass are unlikely to be the cause. The HPLC and MPMC used the same raw chicken slurry compared to chicken meal used in MET. Raw meat has been shown to have higher creatine, which is rapidly and irreversibly converted to creatinine when cooked, compared to cooked chicken meal (Dobenecker and Braun, 2015), aligning with the higher creatinine in HPLC and MPMC compared to MET. However, HPLC had a higher inclusion of the raw chicken slurry, so it would be expected that creatinine would be higher in dogs fed HPLC. It is possible that variation in batch of the chicken and processing of the diets played a role in this small disparity between HPLC and MPLC. While both urea and creatinine can be measures of gross kidney function (Médaille et al., 2004), they all remained within reference range on all treatments.

In conclusion, this study represents the first to assess EE, serum fatty acids and gross measures of health in adult large breed dogs consuming a HPLC diet for 6 wk. All health parameters remained within a normal reference range, suggesting no detrimental effects. In contrast, consuming a HPLC diet led to potentially beneficial effects of increased postprandial EE and fat oxidation compared to the control MPMC diet, which could be effective in managing obesity in dogs, a common health concern, if food intake was appropriately restricted. While pro-inflammatory fatty acids (total n6: n3 and ARA: EPA) were greater in dogs fed HPLC, more research should be done in terms of the effects on pro-inflammatory cytokines, preferably with dogs with an immune challenge or obese dogs in order to investigate the differences in fatty acid metabolism in dogs with inflammation.

## Supplementary Material

txaf018_suppl_Supplementary_Table_S1
