# Supplementary material for: Feeding of a high protein, low carbohydrate diet leads to greater postprandial energy expenditure and fasted n6: n3 fatty acid ratio in lean, adult dogs compared to a moderate protein, moderate carbohydrate diet
Source: Transl Anim Sci. 2025 Feb 6;9:txaf018. doi: 10.1093/tas/txaf018 (PMC11884651; doi:10.1093/tas/txaf018)
Supplement: txaf018_suppl_Supplementary_Table_S1 [file txaf018_suppl_supplementary_table_s1.docx]

**Supplementary Table 1. Effect of diet (high protein, low carbohydrate (HPLC), moderate protein, moderate carbohydrate (MPMC) and metabolic (MET)) on complete blood count parameters in dogs (n=9) at the end of six weeks of feeding.**

| Parameter, unit | Trt | | | SEM | P-value | Reference interval^1^ |
| --- | --- | --- | --- | --- | --- | --- |
|  | HPLC | MPMC | MET |  |  |  |
| White blood cells, × 10^9^/L | 6.1 | 6.3 | 6.3 | 0.498 | 0.740 | 4.9 - 15.4 |
| Red blood cells, × 10^12^/L | 7.5 | 6.8 | 7.2 | 0.329 | 0.344 | 5.8 - 8.5 |
| Hemoglobin, g/L | 179 | 164 | 172 | 7.917 | 0.497 | 133 - 197 |
| Hematocrit, L/L | 0.49 | 0.44 | 0.47 | 0.021 | 0.383 | 0.39 - 0.56 |
| Mean corpuscular volume, fL | 65 | 65 | 65 | 1.547 | 0.962 | 66 - 75 |
| Mean corpuscular hemoglobin, pg | 24 | 24 | 24 | 0.570 | 0.700 | 21 - 25 |
| Mean corpuscular hemoglobin count, g/L | 370 | 372 | 369 | 11.178 | 0.927 | 321 - 360 |
| Red cell distribution width, % | 12 | 13 | 12 | 0.330 | 0.114 | 11 - 14 |
| Platelets, × 10^9^/L | 146 | 148 | 159 | 8.652 | 0.337 | 117 - 418 |
| Mean platelet volume, fL | 10 | 11 | 10 | 0.335 | 0.085 | 7 - 14 |
| Plateletcrit, % | 0.15 | 0.16 | 0.16 | 0.010 | 0.310 | 0.14 - 0.47 |
| Total serum protein, g/L | 73 | 72 | 72 | 1.628 | 0.773 | 55 - 75 |
| Seg Neutrophil Count, × 10^9^/L | 3.1 | 3.5 | 3.3 | 0.431 | 0.650 | 2.9 - 10.6 |
| Lymphocyte Count, × 10^9^/L | 2.4 | 2.3 | 2.4 | 0.256 | 0.825 | 0.8 - 5.1 |
| Monocyte Count, × 10^9^/L | 0.3 | 0.2 | 0.3 | 0.046 | 0.066 | 0.0 - 1.1 |
| Eosinophil Count, × 10^9^/L | 0.23 | 0.37 | 0.32 | 0.075 | 0.207 | 0.08 - 1.33 |

^1^Animal Health Laboratory, University of Guelph, ON. Values established using 86 clinically healthy, adult dogs of various breeds.
